# Rapidly Resolving Myositis Ossificans in an Adolescent Female

**DOI:** 10.7759/cureus.95562

**Published:** 2025-10-28

**Authors:** Nikolaos Laliotis, Panagiotis Konstantinidis, Chrysanthos Chrysanthou, Anastasios Margaritis, Sotiris Sotiriou, Anastasia Morfesi, Georgios Arsos

**Affiliations:** 1 Orthopaedics, Interbalkan Medical Center, Thessaloniki, GRC; 2 Occupational Therapy, University of Western Macedonia, Kozani, GRC; 3 Orthopaedics and Traumatology, Interbalkan Medical Center, Thessaloniki, GRC; 4 Anatomy and Surgical Anatomy, Aristotle University of Thessaloniki, Thessaloniki, GRC; 5 Radiology, Medical Diagnostic Center YGEIA, Veria, GRC; 6 Pathology, Aristotle University of Thessaloniki, Thessaloniki, GRC; 7 Nuclear Medicine, Aristotle University School of Medicine, Papageorgiou General Hospital, Thessaloniki, GRC; 8 Nuclear Medicine, Aristotle University of Thessaloniki, Thessaloniki, GRC

**Keywords:** benign tumor adolescent, gluteal region, heterotopic ossification, myositis ossificans, rapidly regressing

## Abstract

Myositis ossificans (MO) is a rare benign tumor characterized by heterotopic ossification of soft tissues. While the term “myositis” suggests that the lesion commonly affects muscles, it can also occur in rare locations such as tendons or subcutaneous tissues. MO is infrequent in children, primarily affecting young adults and adolescents. It presents distinct radiological and histological features. However, it is crucial to exclude malignancy when investigating heterotopic ossification. Surgical excision of the lesion following an appropriate biopsy is an option, but cases of self-limiting MO have been reported.

We report a unique case of a 14-year-old girl who presented with painful restriction of movement in the lumbar and gluteal regions, without any history of trauma. The girl underwent thorough clinical and radiological investigations, with MRI revealing a circumscribed ossifying lesion measuring 3.8 × 2.3 cm in the gluteal subcutaneous area, lacking clear boundaries, with edema of the adjacent muscles. The lesion exhibited heterogeneous signal intensity and contrast enhancement, while 18F-FDG PET/CT revealed increased metabolic activity within the lesion. A US-guided needle biopsy was performed, confirming the diagnosis of MO with a typical zonal phenomenon. The girl was referred for surgical removal of the lesion within three months after the initial MRI. Upon re-evaluation, the mass was barely palpable, and subsequent MRI and CT scans showed a marked reduction in lesion size. We followed the patient, and in another three months, the mass had almost completely regressed.

We aimed to report a unique case of an adolescent with an established diagnosis of MO, following appropriate radiological and histological evaluation. The rapid regression of the lesion suggests that MO can be managed conservatively in selected cases rather than through immediate surgery.

## Introduction

Myositis ossificans (MO) is a rare benign lesion characterized by heterotopic ossification (osseous and cartilage formation) of soft tissues. Three types of MO have been described: fibrodysplasia ossificans progressiva, traumatic MO circumscripta, and non-traumatic pseudomalignant MO. Although the condition most commonly affects muscle tissue, hence the term “myositis,” it can also occur in less typical locations, such as tendons or subcutaneous tissue [1-4].

While MO has characteristic radiological and histological features, careful evaluation is essential to rule out malignancy when investigating heterotopic ossification, especially when the lesion demonstrates increased metabolic activity on 18F-FDG PET/CT [5-7].

Management often includes observation, as cases that resolve spontaneously have been reported. However, in symptomatic cases, surgical excision may be considered, following a confirmatory biopsy to establish the diagnosis and exclude malignancy [8-11].

We report a unique case with rapid regression within six months. Our patient is a 14-year-old girl who presented with painful restriction of movement in the lumbar and gluteal regions, without any history of trauma. The girl underwent thorough clinical and radiological investigations, including MRI, CT scan, and PET/CT scan, revealing a circumscribed osseous lesion measuring 3.8 × 2.3 cm in the gluteal subcutaneous area, lacking clear boundaries. A US-guided needle biopsy was performed, confirming the diagnosis of MO. The girl was referred for surgical removal of the lesion within three months after the initial MRI. Upon re-evaluation, the mass was barely palpable, and subsequent MRI revealed a significant reduction in size. Follow-up over the next three months showed that the lesion had almost completely resolved.

We want to draw attention to MO in young adolescents, where an established diagnosis of MO can be managed conservatively rather than by immediate surgical procedure, as a primary approach.

## Case presentation

**Figure 1:**
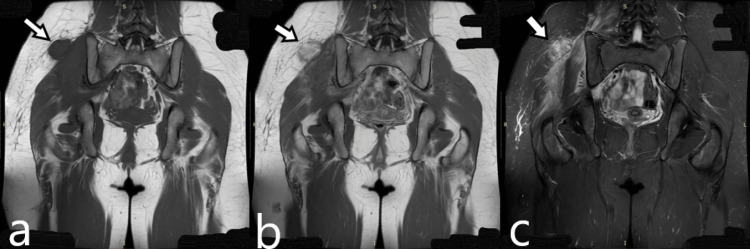
Coronal MRI images (a) Coronal T1W MRI shows a mass in the subcutaneous tissue with signal intensity similar to that of skeletal muscle, without clear boundaries and in contact with the gluteus muscle, measuring 3.8 × 2.3 cm. (b) Coronal contrast-enhanced T1W MRI shows an intensely heterogeneous enhancement of the mass. (c) Coronal FSE STIR MRI shows marked edema of the gluteus medius and minimus muscles and less intense edema in the paravertebral muscles that had the normal striation. FSE, fast spin echo

A healthy 14-year-old girl reported pain in the lower lumbar spine radiating occasionally to the right buttock in mid-October. The girl occasionally participated in handball activities but had not sustained any specific injury preceding the onset of symptoms. After a week of rest and oral paracetamol administration, the pain remained unchanged. The local orthopedic surgeon noted restricted movement in the lower lumbar spine. The girl herself palpated a painful mass in the right gluteal region, prompting an MRI of the lumbar and gluteal areas. The MRI revealed a mass without clear boundaries in the subcutaneous tissue and in contact with the gluteus muscle, measuring 3.8 × 2.3 cm. The mass was circumscribed but lacked well-defined margins, showing heterogeneous signal intensity and asymmetrical post-contrast enhancement. There was marked edema of the gluteus medius and minimus muscles, with less intense edema in the paravertebral muscles, which retained their normal striation (Figure 1).

**Figure 2:**
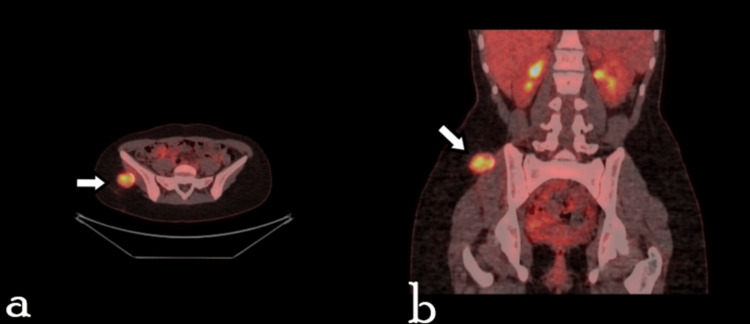
Transverse and coronal 18F-FDG PET/CT images show a hypermetabolic lesion within the adipose tissue, in contact with the gluteal muscle (a) Maximum intensity projection image shows a solitary hypermetabolic lesion within the soft tissues of the right gluteal region, along with axial CT (in bone and tissue windows), PET, and PET/CT sections. (b) The same sections shown in coronal view. The SUVmax of the lesion was 6.6, and the radiodensity measured 42 and 225 HU in the interior and at the calcified rim of the lesion, respectively (for comparison, the radiodensity of the cortical iliac bone was 560 HU).

The girl was referred to a general hospital, where a series of blood examinations was performed, including hematocrit, hemoglobin, total blood count, calcium, phosphorus, CRP, sedimentation rate, and alkaline phosphatase. All results were within normal values. The girl was further referred to a central hospital for additional investigations. A PET/CT scan was performed to evaluate the metabolic activity of the lesion. An 18F-FDG PET/CT scan conducted in November 2024 revealed a 3.3 × 2.7 cm hypermetabolic lesion with a maximum standardized uptake value (SUVmax) of 6.6, featuring a thin peripheral calcific rim, located at the level of the right iliac bone within the adipose tissue in contact with the gluteal muscle. No other hypermetabolic foci were identified elsewhere. Although the metabolic activity raised mild concern for malignancy, the imaging pattern was not suggestive of an aggressive neoplasm (Figure 2).

**Figure 3:**
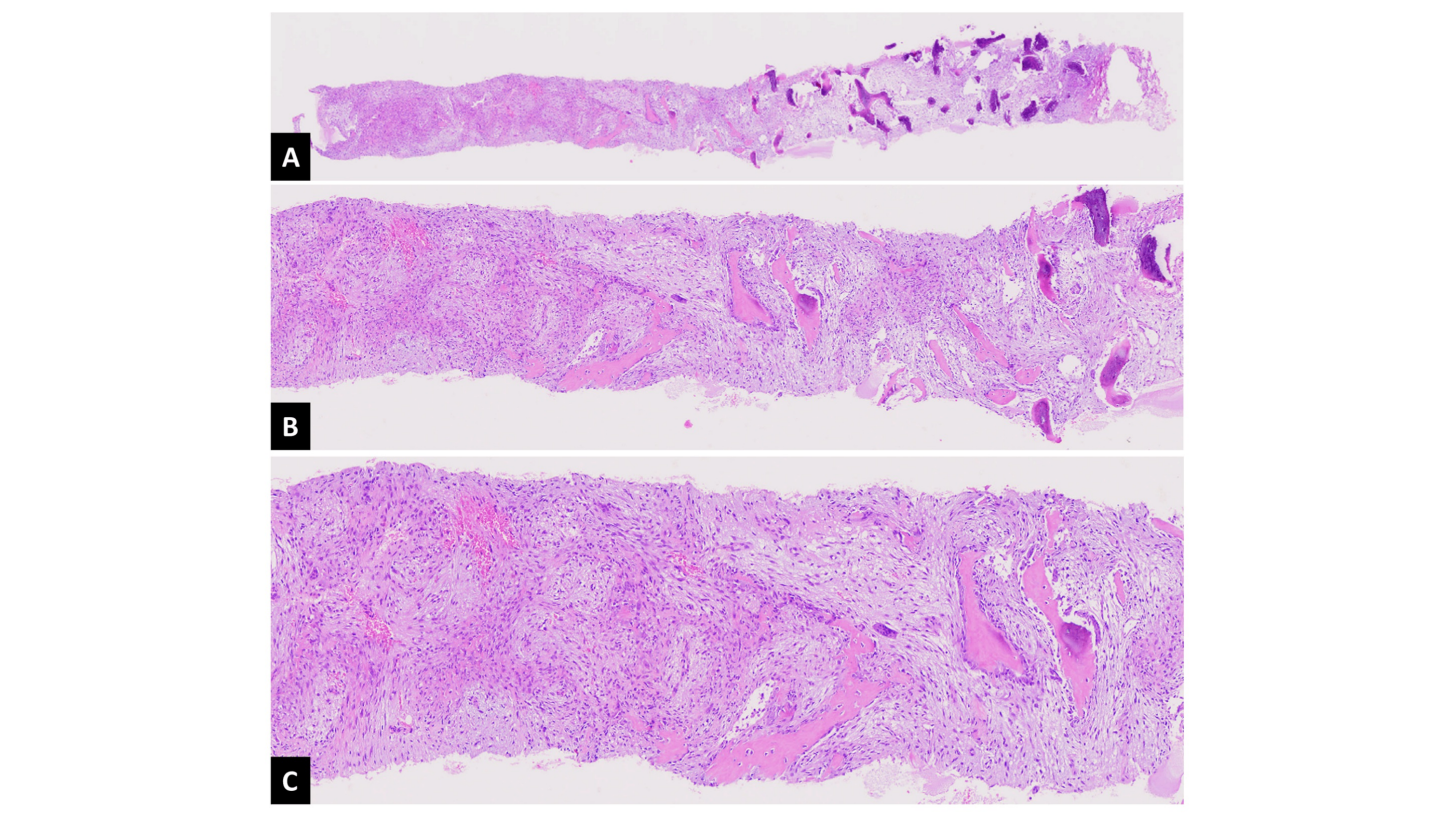
(A) Low-, (B) medium-, and (C) high-power views from the tissue obtained for biopsy, showing the characteristic zonal pattern At the center of the lesion, highly cellular, immature fibroblastic/myofibroblastic tissue is observed within an edematous stroma containing focal hemorrhage and fibrin. Osteoid formation is seen at the periphery, with calcification in the outermost areas (hematoxylin and eosin stain).

The girl underwent a US-guided biopsy that yielded sufficient tissue demonstrating the characteristic zonal pattern, reflecting varying degrees of cellular maturation. The central area of the lesion was highly cellular and composed of immature fibroblastic/myofibroblastic tissue. The fibroblasts/myofibroblasts were arranged in short fascicles or loosely within an edematous stroma, often with focal hemorrhage and fibrin. Osteoid formation was present at the periphery and underwent calcification. The diagnosis of MO circumscripta was established (Figure 3).

**Figure 4:**
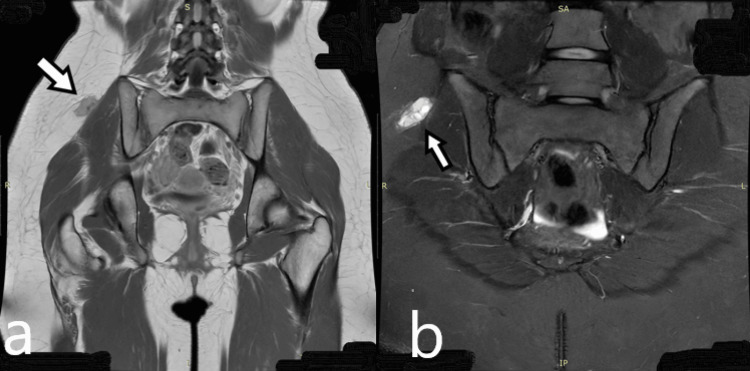
MRI images after two months (a) Coronal contrast-enhanced T1W MRI after two months from the initial MRI shows shrinking of the lesion, measuring 2.1 × 1 cm. (b) Coronal FSE STIR MRI obtained two months after the initial MRI shows reduced edema in the gluteal and paravertebral muscles. FSE, fast spin echo

Three months later, the girl was referred to our pediatric orthopedic department to proceed with the surgical removal of the lesion. Upon examination, we noted a healthy young girl who immediately stated that her pain had subsided. She exhibited normal hip movements and normal flexion and extension of the lumbar spine. The most striking feature was the inability to palpate the previously described mass in the right gluteal region. We requested another MRI, which was performed two months after the initial MRI. The follow-up MRI revealed a reduction in lesion size to 2.1 × 1 cm, with marked improvement in the gluteal muscle edema (Figure 4).

**Figure 5:**
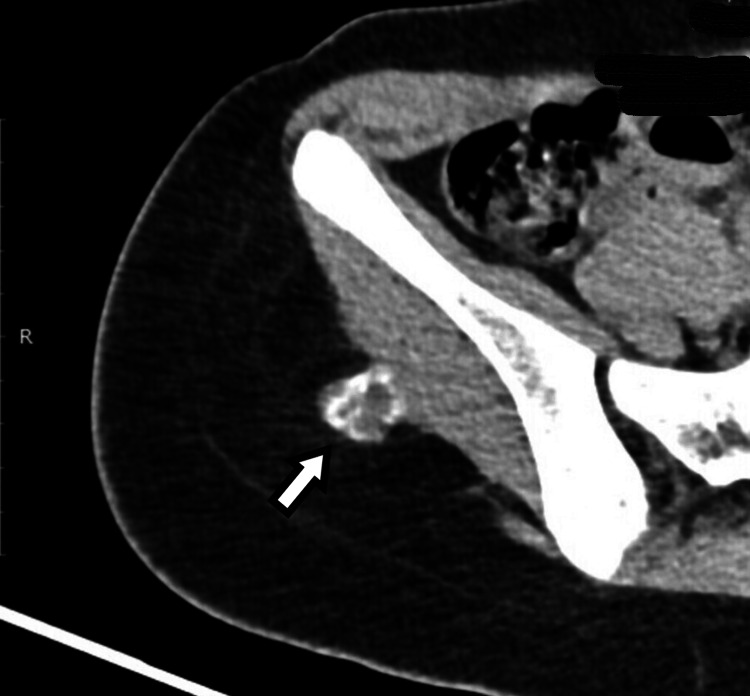
Axial CT scan after two months from the initial MRI shows a mass with peripheral ossifications

We performed a CT scan of the region, which confirmed the small size of the mass with some peripheral calcification. We advised the girl to continue resting and to avoid any vigorous sports (Figure 5).

**Figure 6:**
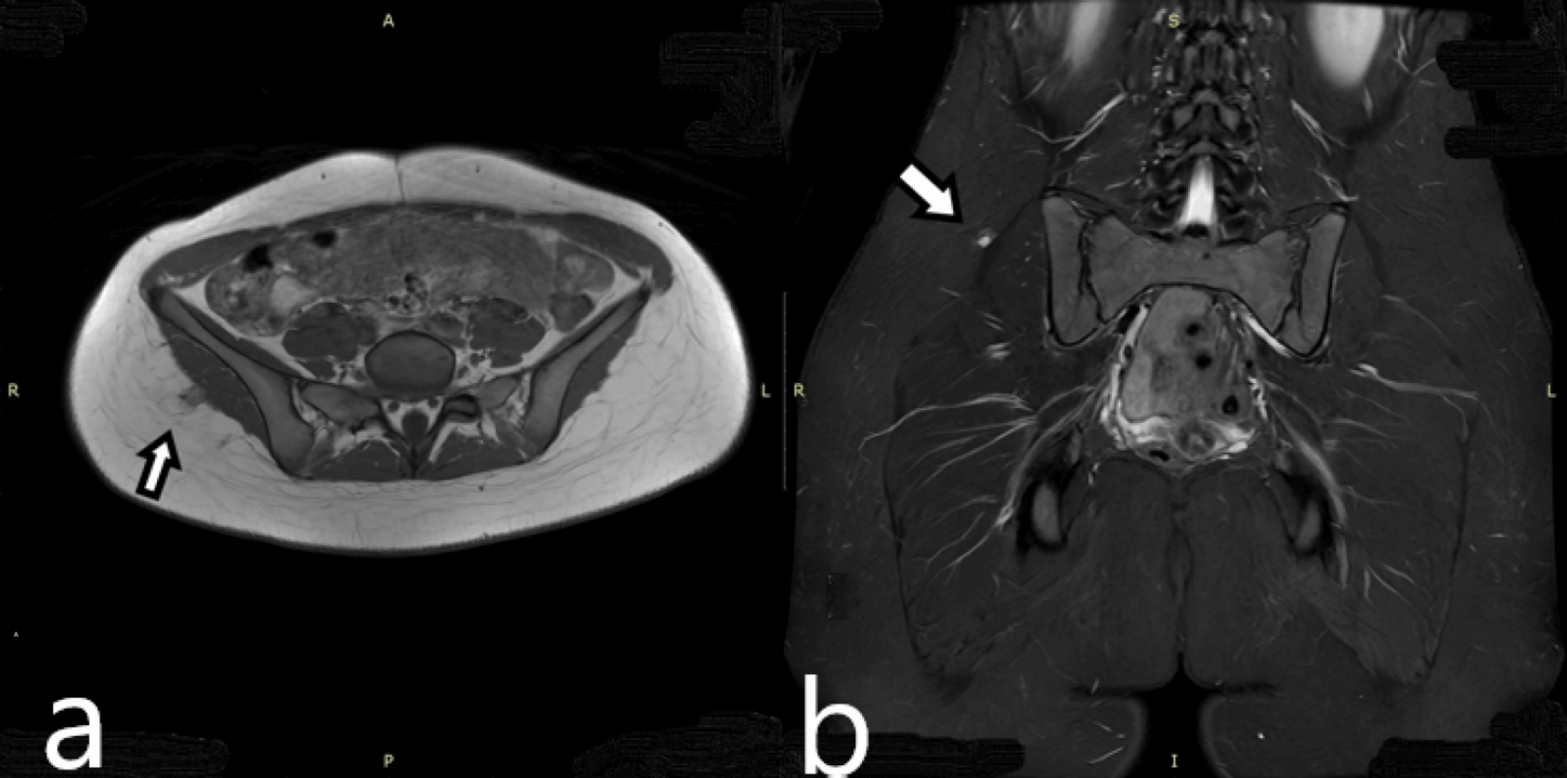
Axial contrast-enhanced T1W MRI and coronal FSE STIR images, after six months from the initial MRI, show a small lesion measuring 0.9 × 0.8 cm, with normal appearance of the surrounding muscles (a) Axial contrast-enhanced T1W MRI. (b) Coronal FSE STIR. FSE, fast spin echo

A new MRI conducted in May barely detected the small lesion, measuring 0.9 × 0.8 cm, with normal muscle appearance. The girl is now participating in her normal daily activities and remains under clinical evaluation at six-month intervals (Figure 6).

## Discussion

Ill-defined lesions within the subcutaneous tissue, showing moderate contrast enhancement and lacking clear boundaries, present a challenging diagnostic problem. MO can mimic malignant lesions in both clinical presentation and imaging, particularly when associated with increased metabolic activity on FDG PET/CT. This case demonstrates a hypermetabolic lesion (SUVmax, 6.6) in an adolescent patient, raising concern [3,4,7,11].

Malignancy, such as extraosseous Ewing sarcoma or osteosarcoma, must be ruled out. Subcutaneous calcinosis and subcutaneous extraskeletal fibroma are among the rare lesions included in the differential diagnosis. Additionally, rhabdomyosarcoma or synovial sarcoma may present as irregular calcifications, primarily in muscle tissue [2,3,12].

MO is rarely found in children. Previous reports have described cases in which MO developed following prolonged immobilization, with gradual resolution over the course of one year [8]. MO predominantly affects muscle tissue, as the name indicates. However, reports of MO affecting tendons and subcutaneous tissues have been reported [2].

MRI investigation is the primary imaging modality that presents features suggestive of MO, but it does not always confirm a definite diagnosis. A striking feature in our patient was the edema of the paravertebral and gluteal muscles. Although there was no clear history of trauma, unrecognized or minor injuries are common in active children participating in sports or physical activities. We suggest that this represents a case of traumatic MO, accompanied by muscle edema secondary to soft tissue injury. The parallel resolution of both the lesion and the muscle edema supports this interpretation. Marked muscle edema is a common finding in MO in the early stages and is rarely observed in cases of sarcoma. This is an important feature for the initial diagnosis [13].

MO typically lacks continuity with the adjacent bone. In cases of traumatic origin, an initial periosteal reaction may appear as part of a periosteal hematoma prior to calcification of the MO. As the MO matures, the separation from the bone becomes evident. Mature lesions are characterized by dense peripheral calcification, a hallmark radiological feature of the condition [4,5,13]. Multimodality imaging is essential, as each modality (US, MRI, CT scan, and PET/CT scan) contributes unique diagnostic features that aid in establishing an accurate diagnosis [11,14].

Although 18F-FDG PET/CT is frequently used in the evaluation of musculoskeletal lesions, high FDG uptake is not specific for malignancy. Inflammatory and reactive processes, including MO, can demonstrate equally high or even higher uptake, as seen in this case. However, since 18F-FDG PET shows a high negative predictive value, metabolically inactive lesions are highly unlikely to represent malignancy. Moreover, as PET/CT is usually performed as total body imaging, the solitary or multiplicity of the lesion(s) is assured [15,16]. 

Histological evaluation is of paramount importance. The term “pseudomalignant” underscores the necessity of accurate diagnosis to exclude malignant lesions. It is crucial to obtain tissue from the entire lesion to confirm the zonal pattern. A biopsy of only part of the lesion may resemble malignancy, leading to inappropriate management [1,4].

MO is characterized by zonal architecture. Initially, it is densely cellular, composed of spindle cells oriented randomly or in short intersecting fascicles. The spindle cells exhibit eosinophilic cytoplasm and plump vesicular nuclei with nucleoli. Normal mitoses are often numerous. The stroma is vascular and myxoid, containing fibrin, extravasated erythrocytes, scattered lymphocytes, and osteoclast-like giant cells. Blood-filled cysts may be present. Peripherally, the spindle cells merge with osteoblasts that rim and populate ill-defined trabeculae and sheets of unmineralized woven bone, which is surrounded by well-formed trabecular and cortical-type bone that remodels into lamellar bone. Occasionally, the matrix contains cellular hyaline cartilage, or the zonal architecture is not well developed. The bone is randomly distributed in MO. An important differential diagnosis is extraskeletal osteosarcoma, which lacks zonation and exhibits malignant cytology [1,4,11].

Cherry et al. reported that a biopsy in the early inflammatory phase may be misleading for the confirmation of MO [4]. In our patient, who was in the early stages with muscle edema, the biopsy provided an accurate diagnosis [4]. MO is often self-limited and may resolve without surgical intervention. NSAIDs are effective in MO management by reducing inflammation and limiting ossification. We have previously reported the regression of MO in children affected in the hip region who were diagnosed with MO after a period of immobilization in the ICU [8].

Sapire et al. reported a similar case of MO in the gluteus maximus of a 13-year-old girl, with a mass measuring 3.4 × 2.5 cm. After radiological investigation and biopsy, she was treated with oral indomethacin, resulting in a marked reduction over a six-month period, with resolution of the edema and a small reduction in the size of the MO, measuring 3.1 × 2cm. In contrast, in our patient, we report the concomitant subsidence of muscle edema and the impressive reduction of the ossification mass in less than six months [17].

Surgery is the primary option for circumscribed MO. It is well established that removal of the lesion cures mature types of MO. Surgery is also anticipated in cases where the diagnosis from biopsy is unclear [18]. Vitale et al. described the surgical removal of MO from the neck of a 12-year-old girl, where radiological and biopsy assessments could not conclude a definitive diagnosis [12]. Rehman et al. performed surgical removal of a mass in the axilla of another 12-year-old girl, where biopsy suggested myxoma, but the surgical specimen revealed MO [11]. Isse et al. reported the removal of an MO from the abdominal wall in a seven-year-old child, which was located in the subcutaneous area, after three years from the initial presentation [19]. Surgical treatment is the choice when there is pressure on nerves or vessels. Cherry et al., in a recent review of 60 pediatric cases of MO in patients aged 0.2-17 years, reported that 47% of the patients underwent surgical treatment [4].

We have not found any previously described cases of rapidly resolving MO occurring within less than six months.

## Conclusions

This case report indicates the importance of considering MO in the differential diagnosis of ossifying lesions of soft tissue in children and adolescents. It is essential to perform multimodality investigations, as extraosseous ossified lesions pose significant diagnostic challenges. Recognizing characteristic features on MRI and PET/CT scans, along with the zonal phenomenon on histology, can guide clinicians toward an accurate diagnosis. An established diagnosis allows for conservative management and observation rather than performing early surgical procedures based on presumed malignancies. This case illustrates the potential for rapid spontaneous regression of both the lesion and the associated muscle edema, emphasizing the value of clinical and radiological follow-up in selected cases.
